# Supplementary material for: Effects of copper nanoparticles synthesized from the entomopathogen Metarhizium robertsii against the dengue vector Aedes albopictus (Skuse, 1894)
Source: PLoS One. 2024 Nov 25;19(11):e0314279. doi: 10.1371/journal.pone.0314279 (PMC11588212; doi:10.1371/journal.pone.0314279)
Supplement: Attachment — Submitted filename: Response to Reviewers Comments.docx [file pone.0314279.s002.docx]

**Journal Requirements:**

Please review your reference list to ensure that it is complete and correct. If you have cited papers that have been retracted, please include the rationale for doing so in the manuscript text, or remove these references and replace them with relevant current references. Any changes to the reference list should be mentioned in the rebuttal letter that accompanies your revised manuscript. If you need to cite a retracted article, indicate the article’s retracted status in the References list and also include a citation and full reference for the retraction notice.

- **Thank you, professor, for your valuable information, as per your information the retracted reference has been removed and new reference has been replaced in the revised manuscript. Thank you.**

**Reviewer #1:** 
Thank you for considering my comments. However, the following issues remain open.
What does ppm/ml mean? Normally, the concentrations should be expressed in ppm of CuNPs test solution to water solution and the metric units of ppm should be clarified, e.g., mg/lt or μl/lt. Please check.

- **Thank you, professor, for your comments, as per your comments we corrected the errors in the revised manuscript. Thank you.**

You did not reply to my answer on how you are sure that copper does not contribute to the effect of copper nanoparticles of fungi to mosquitoes.

- **Dear professor, I did not specifically claim that copper does not contribute to the effect. However, the unique properties of nanoparticles, such as surface area and reactivity, significantly differ from bulk copper's effects on mosquitoes.**

In the text it should be clarified that for the adulticidal toxicity an untreated control was also used and specify the replicates used.

- **Thank you, professor, as per your comments, the missing replicate information’s has been added in the revised manuscript.**

Your response about the table with LC values is noted. However, I suggest adding in the text the confidential limits in parenthesis next to each LC50 or LC90 value.

- **Thank you, dear professor, as per your comment we have added the upper and lower confidential limits has been added in the revised manuscript. Thank you**

**Reviewer #2:** 
The authors have responded to all the reviewer comments, and the manuscript now meets scientific standards. This revised manuscript is well-written and presents a novel study that contributes significantly to the field. The methodology is sound, and the data are clearly presented with appropriate analysis. The findings are both original and impactful, and the discussion effectively integrates the results within the broader context of existing literature. I now recommend this revised manuscript for publication.

- **Thank you, Professor, for reviewing our research manuscript and for your valuable comments and suggestions. Your feedback has been incredibly helpful in improving the quality of our manuscript. We truly appreciate your kind support.**
