# Supplementary material for: Effects of copper nanoparticles synthesized from the entomopathogen Metarhizium robertsii against the dengue vector Aedes albopictus (Skuse, 1894)
Source: PLoS One. 2024 Nov 25;19(11):e0314279. doi: 10.1371/journal.pone.0314279 (PMC11588212; doi:10.1371/journal.pone.0314279)
Supplement: Attachment — Submitted filename: Response to Reviewers Comments.docx [file pone.0314279.s003.docx]

**Journal Requirements:**

Please review your reference list to ensure that it is complete and correct. If you have cited papers that have been retracted, please include the rationale for doing so in the manuscript text, or remove these references and replace them with relevant current references. Any changes to the reference list should be mentioned in the rebuttal letter that accompanies your revised manuscript. If you need to cite a retracted article, indicate the article’s retracted status in the References list and also include a citation and full reference for the retraction notice.

- **Dear Sir/Madam,**

**In response to your comments, we have thoroughly checked the references and can confirm that no retracted articles have been cited. However, if you identify any references that have been retracted, please let us know, and we will promptly remove and replace them with recent and relevant citations.**

**Thank you for your assistance.**

**Additional Editor Comments:**

The authors need to revise and fix the used units in the manuscript.

- **Thank you, Professor, for your valuable comments. In line with the reviewer’s feedback, the errors have been corrected in the revised manuscript. I appreciate your guidance and support.**

**Reviewer #1**

Thank you for your reply. One last point is still open. Please indicate in the materials & methods of the manuscript the metric units of the tested material (e.g., in mg/L or μl/L) that are expressed in ppm.

- **Thank you, Professor, for your valuable comments. Your feedback has been extremely helpful in strengthening our manuscript. As per your suggestions, the test concentration units have been added in the revised version. Once again, thank you for your support.**
